# Children’s Microsystems and Their Relationship to Stress and Executive Functioning

**DOI:** 10.3389/fpsyg.2020.00996

**Published:** 2020-07-30

**Authors:** Jose Antonio Campos-Gil, Patricia Ortega-Andeane, Delfino Vargas

**Affiliations:** ^1^School of Psychology, National Autonomous University of Mexico, Mexico City, Mexico; ^2^University Program of Development Studies, National Autonomous University of Mexico, Mexico City, Mexico

**Keywords:** microsystems, executive functions, children, classroom, stress

## Abstract

Microsystems are described as contexts formed by a subject, their roles, their interactions, and a specific physical space and time, such as housing and the school environment. Although several studies suggest the importance of studying this type of environment and its repercussion on children’s development, in a Latin American context, few studies integrate the interaction of two primary settings in the development of executive functioning. The present study explores the effects of the quality of housing and school environments on the perception of stress, decision making, and planning among children. A total of 114 children (43% girls and 57% boys, *M*_age_ = 10.57) from a primary school located in a community classified as poor participated in the study. The following was measured: the environmental quality of classrooms, housing, stress, and executive functioning of children. The results reveal a model linking environmental quality levels in children’s homes and schools and executive functioning. We also obtain a mediating role of stress between microsystems and performance, finding a deficit in executive performance when children experienced higher levels of stress as a result of poor environmental quality both in their homes and in their schools.

## Introduction

Although studying the effects of environmental settings on cognitive development has become in recent years a field of extensive research in various domains such as economics (World Bank, 2014), psychology (Montero and Evans, 2010), and epidemiology (Garcia-Coll et al., 1998), it is still to be studied comprehensively in Mexico, although the specific context makes the topic a national priority.

Indeed, according to the latest report of the National Council for the Evaluation of Social Development Policy in Mexico (CONEVAL), the percentage of people in poverty is 43.6% of the total population (CONEVAL, 2013; CONEVAL, 2015). According to the National Institute of Geography and Statistics (INEGI, 2011), by 2015, 27.26% of the population was between the ages of 0 and 14, representing a large group in the country; of these, 21.4 million were children and teenagers. The United Nations Children’s Fund (UNICEF, 2015) reported that 4.6 million were in extreme poverty, making children one of the most vulnerable groups, who experience deprivation throughout their development (UNICEF, 2015). These deprivations can be harmful, in health, including even physical deprivation in their main environments.

Given the magnitude of the phenomenon and the context in which these children and young people are immersed, it is necessary to extend systematic research on the effect of the physical and psychosocial elements involved in housing, schools, and neighborhoods (Lawrence, 2010).

At present, it is understood that child development is influenced by not only epigenetic factors but also environmental factors. Various investigations have found evidence of the impact of the socio-physical environment on the life span of a child, within the first years of life (Ferguson et al., 2013), especially if we refer to the family environment, which is the first microsystem with which the child interacts.

Similarly, Maxwell (2009, 2016) provided evidence on the importance of educational settings, such as the number of students and teachers and the available space for occupation within the school. Indeed, the performance and well-being of both students and teachers are affected by overcrowding, interestingly not only within the classroom but also in the entire school environment. Maxwell considers the role of the planning and control of enrollment, the size of classrooms, the arrangement of furniture, and the rotation of teachers. She finds that all these elements tend to generate environmental inconsistencies that stress the children. Similarly, the negative impact of noise near the school has been established, not only because of the stress and hearing impairment it produces but also because of the difficulty in concentration and understanding and its impact on problem solving and decision making (Evans et al., 2005; Evans, 2006; Estrada, 2007).

### Bioecological Model of Development

Bronfenbrenner (1986) offers a theoretical perspective for human development and especially of the evolving interaction between the person and ecological environment, conceived as a set of nested structures, each inside the next, like a set of Russian dolls. As Bronfenbrenner (1979) said, “at the inner most level is the immediate setting contained developing person this can be the home or the classroom” (p. 3). A system is understood as a set of elements formed by a day, roles, a process, a socio-physical context, and time. The first is the “microsystem.” The second is the “mesosystem” comprising the interrelationships of two or more environments in which the developing person actively participates, for example, the child and the relationship between home and school, which comprises any alteration in the home that will impact the school and vice versa; for example, the death of a family member at home will affect his or her development at school.

Bronfenbrenner (1986) postulates that development is a mutual and progressive adaptation between human beings and the changing properties of their immediate environments. Therefore, the change of one element in the system will have ramifications in all the others and finally “the development of themselves,” which must be understood as the result of the biopsychosocial alterations of a person throughout his/her life (Bronfenbrenner and Morris, 2006).

Finally, at the final level, learning and psychological functioning are influenced by multiple and nested layers in a *macrosystem*, where such layers or microsystems are connected through the individual, and although these microsystems differ from each other, both affect an individual. Therefore, a second environment could moderate the experiences of the original microsystem. For example, playing an important role here is how children cope with stress, that is, the use of both individual and external resources to cope with problems.

However, it is important to expand the evidence regarding deficits in executive functioning; this construct is responsible for integrating many of the processes that allow us an adequate response to the environment that surrounds us, with other individuals, or an adequate cognitive goal functioning (Hackman et al., 2015).

This is the case of those who live and grow up in poverty, in insecurity, or without access to quality formal education; socioeconomic status (SES) also appears to affect school attendance and the number of years of schooling completed. The impact on years completed appears to be less than the impact on school achievement. Also, low-SES children more often manifest symptoms of psychiatric disturbance and maladaptive social functioning than do children from higher-SES circumstances (Bradley and Corwyn, 2002).

From early childhood onward, the number of settings in which the growing person actively intervenes increases gradually (for instance, home, school, and work). As a consequence, this growing participation is both a cause and a consequence of development (Bronfenbrenner, 1986); interactions must occur on a regular basis and over prolonged periods of time (Bronfenbrenner and Evans, 2000; Coley et al., 2015; Berry et al., 2016). The lack of routines and structure in the home has been found to be negatively associated with psychological adjustment in children, family satisfaction, and school performance (Fiese, 2006; Flouri, 2009; Fennis and Wiebenga, 2015).

### Household Environmental Quality

The housing includes indoor and outdoor areas. Some aspects of housing and health are directly affected by the quality of infrastructure, as well as the conditions of the communities in which they are located (Ferguson et al., 2013). The main health effects include respiratory diseases related to indoor air quality, mortality related to thermal comfort, diseases transmitted due to exposure to pests, airborne infections, waterborne diseases, and domestic injuries. In terms of mental health, the sources are effects from noise, lead poisoning or exposure to other hazardous wastes such as asbestos, poor urban design, high residential density, the immediate environment of the dwelling (insufficient hygiene and sanitation), and type of dwelling.

To promote optimal development, according to the literature (Ferguson et al., 2009; Hackman et al., 2015), the household environment should (1) sustain the child (ensuring those elements that guarantee the biological integrity of the child), (2) stimulate activity in the child (aimed at the improvement of the child), (3) support the child’s self-sustaining capacities and trends, and (4) control the quantity and pattern of experiences for the child.

Caldwell and Bradley (1984) identified five basic functions that are performed by housing: (1) sustenance, (2) stimulation, (3) support, (4) structure, and (5) supervision. For the purposes of this paper, only four are highlighted.

### School Environment

In the particular case of Mexico, not all schools have the possibility of moderating their social density through district mechanisms such as those used, for example, in the United States. In 2006, Mexico had an average of 21 children per classroom in public sector schools, reaching groups of 35 students in some states (INEE, 2006); Maxwell (2006) refers that one way to reduce the perception of overcrowding is by regulating enrollment or by building schools that provide more space per child; however, this implies higher economic costs, making it more feasible to address other variables, such as noise, whose presence in classrooms has been proven to hinder the intelligibility of words in children (Estrada, 2007); also lack of concentration affects not only academic performance but also the development of adequate emotional regulation (Maxwell and Evans, 2000; Zimmerman, 2005; Blair, 2010) and makes it impossible to attend to the content of the classes (Evans and Lepore, 1993).

The school is the space most important after home; initially, the school environment was not considered a relevant variable in the teaching–learning process, but lately, research has sought to break down the elements of the school environment and its impact on children’s development (Evans, 2004; Estrada, 2007; Maxwell, 2009; Abry et al., 2017).

In Mexico, important achievements have been made in terms of coverage of primary education; nevertheless, there are important disparities in education and school settings causing a significant proportion of the poor or most vulnerable sectors to not have access to them and causing many of those who enter education to not be able to conclude their studies (Bulotsky-Shearer et al., 2012; Hackman et al., 2015; Maxwell, 2016). This inequality in supply extends to various states, between rural and urban areas, and to private and public schools (UNICEF, 2015).

Today, UNESCO believes that quality must go beyond ensuring that children enter and remain in the classroom; rather, it is the “combination of teaching–learning conditions and student achievement” (UNESCO, 2013).

Research has revealed the importance of various aspects of the school, each having specific effects on development. First, the availability of school facilities and equipment or the sufficiency (concrete or perceived) of educational facilities, services, furniture, materials, and equipment as a necessary condition for school processes to take place (Comité Administrador del Programa Federal de Construcción de Escuelas [CAPFCE], 2001; NCERT, 2001; INEE, 2006; Walden, 2015). The physical conditions of facilities and equipment, that is, the design, operation, maintenance, and age of facilities, can have a positive impact on student experiences and consequently on educational performance (Bulotsky-Shearer et al., 2012). Research has also shown the importance of physical comfort in the classroom, for example, thermal comfort, ventilation, acoustics, lighting, and the quality of the furniture (Hannah, 2013; Hatfield et al., 2016). Maxwell (2009) argued that a school with high levels of noise, overcrowding, confusion, changes in teachers or classmates, poor-quality infrastructure, or an absence of routines might negatively affect children’s development. In spaces with quality deprivations, it is common for both children and teachers to experience overcrowding, due to density, temperature, and lighting (Maxwell, 2006); a student or teacher may then feel overcrowded when he or she observes that his or her ability to control interaction with others is affected or when others interfere with his or her ability to perform an activity such as reading and studied conversation (Fennis and Wiebenga, 2015).

Indeed, schools with higher enrollment have been identified as having reduced opportunities for meaningful student participation, higher dropout rates, and disruptive behaviors among students (Maxwell, 2006).

In contrast, schools with lower social density, with low-income students (Johnson, 2000), found better student attitudes, greater attention span, fewer behavioral problems, greater participation in extracurricular activities, and greater leadership development (Maxwell, 2006; Buckrop et al., 2016).

### Stress

#### Psycho-Environmental Perspective on Stress

There are a variety of factors that can mediate the reaction of stress for which environmental stress is defined as the product of psychological aspects that arise from the relationship of the individual with the environment; its study is carried out in previously defined contexts, where the dynamics and relationship between the demands of the environment toward an individual and the resources available to face them have been reflected (Baum et al., 1981; Kaminoff and Proshansky, 1982; Evans and Cohen, 1991; Ortega et al., 2016).

#### Environmental Stressors

1. Temperature (Evans and Stecker, 2004; Halpern, 1995): An inverted “U” effect has been observed, indicating that at higher levels of heat, aggressive and hostile behaviors increase with temperature, but this relationship begins to reverse to a certain point where temperature continues to rise.
2. Noise: Noise is a factor that has serious psychological effects on individuals. It is important to specify that noise is that sound that is undesirable and annoying to the listener; therefore, not everyone will experience noise in the same way. It has been found that sounds at high decibels are linked to effects on mental health, interference in tasks, the intelligibility of words, and attention (Evans, 2006; Estrada, 2007).
3. Overcrowding: The subjective perception that available space is insufficient depends on the person’s assessment of the environment which can be independent of density, which is the objective measurement of a person’s physical space, where, for study, social density (the variation of the number of people in a fixed space) is distinguished from spatial density (the variation of space in relation to a number of people) (Lawrence, 2010).

While overcrowding is closely related to both spatial and social density (Maxwell, 2006), a student or teacher may then feel overcrowded when he or she observes that his or her ability to control interaction with others is affected or when others interfere with his or her ability to perform an activity such as reading, studying, or talking.

#### Developmental Stress

Many studies have found that receiving low-quality care at an early age can serve as a primary source of stress for children. Indeed, households more likely to be overcrowded, disorganized, and lacking appropriate stimulation and resources for an adequate quality of life can result in physiological stress markers being found in young people, who had presented a high allostatic load during their childhood (Smith and Ezzati, 2005; Evans and Kim, 2012). This association is mediated by the time of exposure: the longer a child grows under poor physical conditions, the more the cumulative damage increases (Curby et al., 2010).

Garcia-Coll et al. (1998) have evaluated cognitive functions such as memory and problem solving in low-income children. Most longitudinal studies have found that young people who experienced less poverty or had a lower allostatic load during childhood obtained better grades and retained more information than their peers with longer exposure times.

### Executive Functions

Suchy (2009) explained that executive functions (EFs) are neuropsychological constructs that can be defined as the ability to form, maintain, and change mental states; this implies reasoning skills, the generation of goals and plans, the maintenance of concentration, and the flexibility to alternate between those goals and plans in response to contingencies that may arise. Although EFs can be studied from a purely functional approach, considering their anatomical substrate provides valuable information regarding their organization and functioning; it is therefore paramount to clarify that in anatomical terms, the prefrontal cortex (CPF) is a primordial area in charge of EF, by means of which it has been studied that one of these functions have been the establishment of goals and planning, defined as the capacity to reach goals or objectives in the short or long term (Baker et al., 1996; Lozano and Ostrosky, 2011).

### Environment and EFs

The effects of both the natural and constructed environments have been explored in relation to the broad range of functioning, including the cognitive process (Hygge and Knez, 2001), affection (Loewen and Suedfeld, 1992), and health (Evans et al., 2003); among the main settings evaluated have been educational institutions where they have evaluated the amount of light, temperature, and sound and its relationship to learning or predecessor functions to learning (Choi et al., 2016). The main findings contribute to considering noise as an irrelevant stimulus that diverts and even filters the attention that a person pays to a specific goal (Hygge and Knez, 2001; Hannah, 2013); likewise, it has been observed that the absence or interruption of learning resources in the home or school classroom is a predictor of decreased inhibitory control and cognitive flexibility (Hackman et al., 2015).

While the variety of settings in which a human being can grow up is wide, environments that are physically and socially impoverished can have long-term detrimental effects on the child, such as stress and problems in planning, attention, or decision making. It is reasonable to believe that children exposed to multiple stressful contexts experience greater vulnerability compared to children exposed to fewer stressful contexts; therefore, the objective was to explore the relationships among quality of housing and school with the perception of stress, planning, and decision making in children.

## Materials and Methods

### Participants and Setting

To obtain the sample, the research project was presented in a public school in the State of Mexico, and specifically in the municipality of Chalco, where the percentage of poverty is 54.5% and that of extreme poverty is 8.7% and which is classified nationally as one of the municipalities in “average” poverty according to the study by Cortés and Vargas (2019).

In this school, they were interested in the research, so a meeting was held with the director of each institution to establish a work route, which consisted of a round of information to parents, that is, informing in a group session the objectives of the research and requesting consent as well as ensuring the privacy of the information obtained.

The sample consisted of 114 children, of whom 43% were girls and 57% were boys with an average age of 10.57 years (9–11 years old, SD = 0.51), coming from a charitable school which differs from the socioeconomic level of children’s housing because it has a better infrastructure than other schools in the same area. Participants’ house and school were in the poorest community of Valle of Chalco, which has a population of 357,645 (as of 2010), where at least 20% experience educational backwardness, up to 30% has low-quality housing, and up to 50% do not have access to social security (CONEVAL, 2015).

### Instruments

#### Physical Measurements

##### Spatial density

Spatial density was measured with a laser distance meter, and the dimensions of the classroom were then calculated as the area in square meters over the number of students at the time of evaluation.

$$\frac{a\,r\,e\,a}{s\,t\,u\,d\,e\,n\,t\,s}$$

##### Noise

A digital decibel meter (Model HER-403, with a measurement range of 30–130 dB) was used to record the noise levels inside the classroom, calculating the average decibels for 20 min.

##### Temperature

The temperature inside the room was recorded with a digital thermometer with a humidity sensor (Model TER-150).

#### Environmental Variables

**FIGURE 1:**
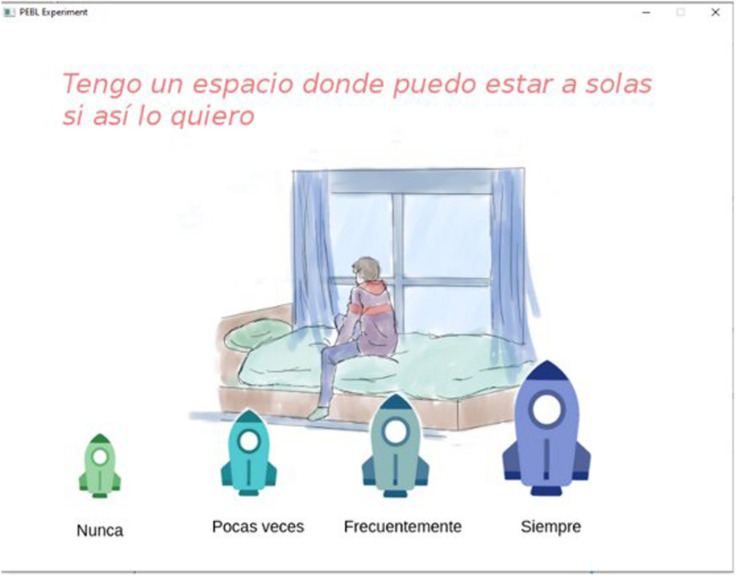
Reactive example of ECAV: “Have a space where I can be alone if I wish?”

The Environmental Quality Scale for Housing (EQSH) that was created for the study consists of 26 items and had a total Cronbach alpha of 0.886. With this scale, participants have to indicate the presence or absence of certain characteristic in their houses with four response options (Never to Always); the items are distributed in four factors: support, stimulation, social integration, and structure (Figure 1).

**FIGURE 2:**
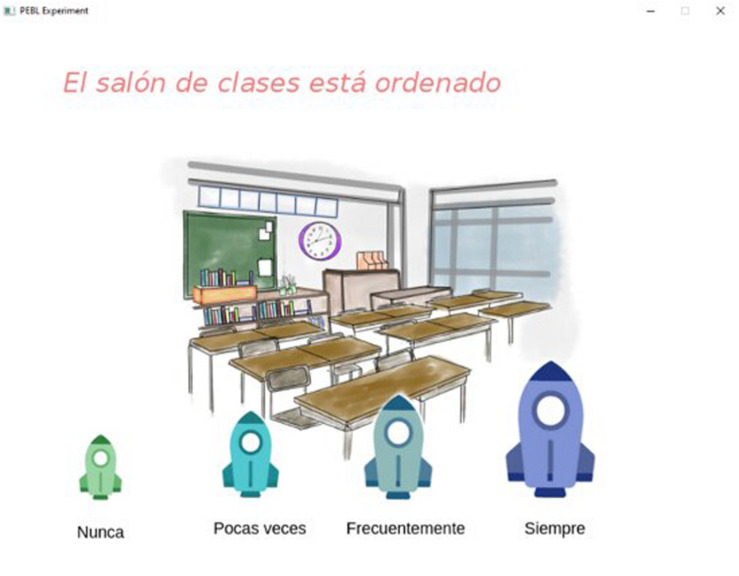
Reactive example of ECAE: “Is the classroom tidy?”

The School Environmental Quality Scale (SEQS) that was created for the study consists of 24 reagents and had a total Cronbach alpha of 0.880. With this scale, participants have to indicate the presence or absence of certain characteristics in schools with four response options coded as 0–3 (Never–Always); the reagents are distributed in four factors: support, stimulation, social integration, and structure (Figure 2).

**FIGURE 3:**
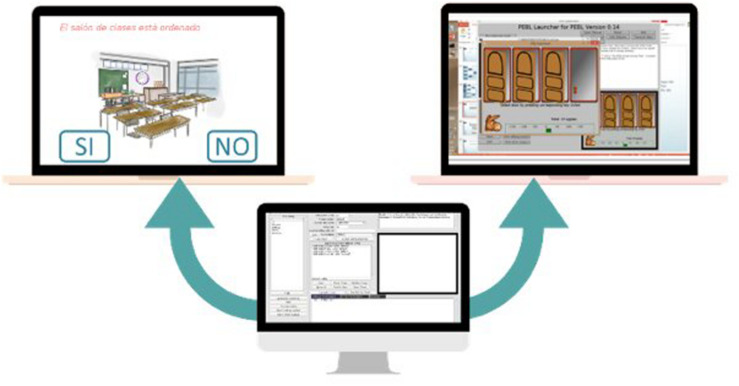
PEBL screen.

For both scales, a graphic representation was designed by item, looking for the illustrations to facilitate the comprehension of the children; these scales were applied through the software PEBL (The Psychology Experiment Building Languaje, Figure 3).

#### Psychological Measure

Child Stress Scale consists of 44 multiple-choice items (“nothing,” “little,” “enough,” and “much”) by means of a pictorial scale; these are grouped into eight factors: lack of family acceptance, verbal aggression and mockery, lack of trust and respect, school pressure, family demand, punishment, family conflicts, and fears. Finally, the scale has a global alpha of 0.91 (Lucio et al., unpublished manuscript).

#### Executive Functioning Tasks

In the case of decision making, the complexity is higher; however, neuropsychologists have focused on working with conditions of ambiguity and uncertainty, using a system of rewards and punishments (Iowa Gambling Task) where the key variable is the degree of consistency in the pattern of decisions (Verdejo-García and Bechara, 2010).

The “Hungry Donkey Task” (HDT) is an isomorphism of the “Iowa Game of Chance” (Mueller and Piper, 2014). The HDT consists of four blocks of 70 trials for a total of 280 trials presented where a donkey chooses from four doors, each with a cost or reward in apples; the task will be that the child plans and makes decisions from the feedback of the doors, the ultimate goal being to give the donkey as many apples as possible (Crone and Van Der Molen, 2004).

In the “Simón Task,” children are presented with congruent stimuli: arrows in the right or left field of vision; these will be pointing to the left or right, so children will respond through the button that goes according to the direction of the arrow. However, there will also be incongruent stimuli: arrows pointing to the left will appear on the right side, and vice versa. The number of trials is 140, which are presented randomly in two sets. The time it takes to run the experiment is about 15 min.

### Procedure

**FIGURE 4:**
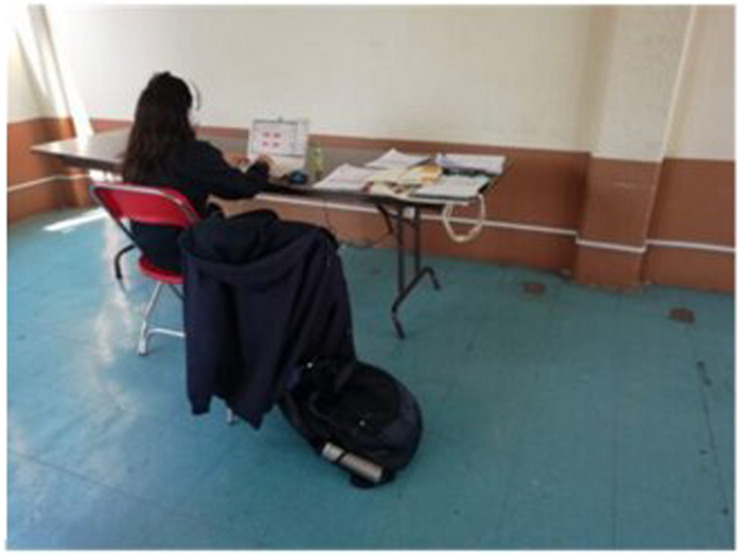
Student responding to computer tests.

The inventories were applied alternating between the application of psychometric instruments (using pencil and paper), followed by those where respondents answered in a computer (ECAV and ECAE) and at the end the HDT and “Simón Task.” Each set of tests was applied on different days, starting with psychometric instruments, followed by the ECAV and ECAE on the second day and the HDT and “Simón task” on the third day, with the purpose of diminishing the fatigue of the evaluation (Figure 4).

Once all the instruments were applied, the information was coded and entered into the statistical program STATA, for the preparation of the database, to finally analyze the data with the Smart PLS data package.

### Plan of Analysis

We opted for a partial least squares (PLS) model in which the focus is on the analysis of variance as opposed to models [structural equation model (SEM)], which worked with covariance for this type of analysis using the software Smart PLS. A partial model is a modeling technique with latent variables, such as the quality of the spaces, which allows the incorporation of multiple dependent constructs, such as the factors that build these scales, like support, stimulation, structure, and social integration, explicitly recognizing the measurement error. However, the least restrictive is in terms of normal distribution assumptions, allowing us to simultaneously estimate/explore both a measurement model and a structural model from a small sample.

## Results

It is important to mention that by internal regulations of the school, the classes are not mixed in terms of gender. There were 114 students who were grouped into four groups according to sex and school year: fifth-year girls (*n* = 14), fifth-year boys (*n* = 32), sixth-year girls (*n* = 35), and sixth-year boys (*n* = 33). The sample was selected intentionally, where the groups were previously formed. We applied the instruments to all the students from the fifth and sixth grades.

In relation to the physical conditions of the classrooms, the following scores were obtained per group (Table 1), finding that the boys’ classrooms were larger (*t* = −85.104, *p* < 0.0001) and at the same time warmer (*t* = −33.732, *p* < 0.0001) than the girls’ classrooms and also noisier (*t* = −2.745, *p* = 0.007) (Table 1).

**TABLE 1:** Physical characteristics of classrooms in the function of the gender and the grade.

	**Fifth grade**		**Sixth grade**	
	**Female (*n* = 14)**	**Male (*n* = 32)**	**Female (*n* = 35)**	**Male (*n* = 33)**
Spatial density	1.42 m^2^	1.69 m^2^	1.34 m^2^	1.82 m^2^
Temperature	22°C	25°C	20°C	24°C
Noise	53 dB	54.2 dB	58.3 dB	62.6 dB
Classroom area	46.86 m^2^	57.78 m^2^	46.72 m^2^	60.07 m^2^

**FIGURE 5:**
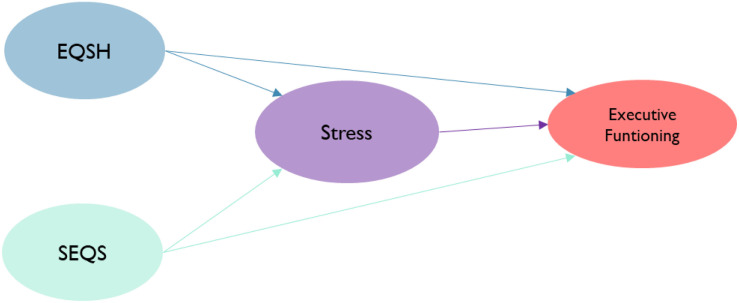
Formative model.

As for the perception of the quality that the children (Table 2) had of their microsystems, although this does not differ from gender, it does so from one school year to another. Both at home and at school, significant differences were found where the fifth-grade group indicates greater support both in their classroom (*F* = 4.53_DF=__78_, *p* = 0.035) and in their homes (*F* = 5.41, *p* = 0.022). On the other hand, sixth-grade children reported that their classrooms provided more of a structure, unlike their younger congeners (Figure 5).

**TABLE 2:** Environmental quality factors by grades.

		**Fifth year**	**Sixth year**	***t***	***p***
School	Social integration	2.25	2.31	–0.578	0.565
	Structure	2.15	2.4	–2.976	0.004
	Support	1.74	1.73	–0.069	0.945
	Stimulation	1.98	2.18	–1.766	0.081
Home	Social integration	1.46	1.64	–1.127	0.263
	Structure	1.75	1.72	0.200	0.842
	Support	2.06	2.23	–1.416	0.160
	Stimulation	1.94	2.03	–0.916	0.362

### Stress

**FIGURE 6:**
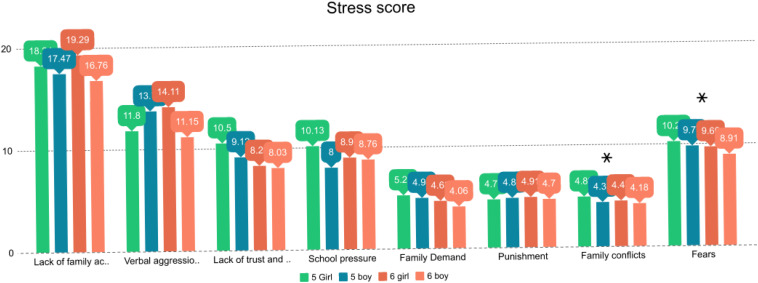
Stress score by gender.

Girls in both fifth (*F* = 6.230, *p* = 0.017) and sixth grades (*F* = 5.526, *p* = 0.022) experienced higher levels of stress compared to boys, related to family conflicts and fear. Compared with boys of the same age, in the rest of factors, although they obtained higher scores, they did not differ enough on statistical terms (Figure 6).

### Executive Functioning

It can be seen in Table 3 that although the groups, in terms of gender, had results in terms of planning below 0, the *t*-test (1,246, *p* = 0.213) indicates that there were no significant differences.

**TABLE 3:** EF scores.

	**Fifth grade**		**Sixth grade**	
	**Female**	**Male**	**Female**	**Male**
Total HDT	−19	−24	−10	−24
Good HDT	67	68	70	66
Congruent Hit	25.28	26.59	21.77	27.09
Incongruent Hit	23.22	23.47	18.36	24.91

**FIGURE 7:**
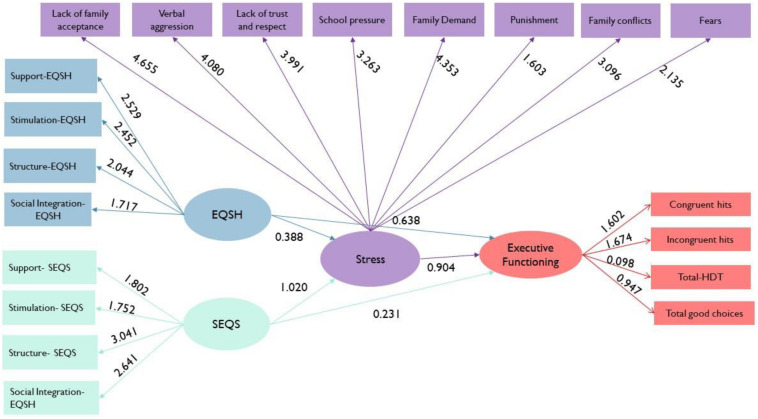
Structural equation model using partial least squares (SEM-PLS).

We fit an SEM-PLS model in this investigation. First, we estimated a second-order factor model for the latent variable called environmental quality of the home EQSH (support, structure, stimulation, and integration). Similarly, eight factors conform to the stress latent variable (lack of family acceptance, verbal aggression, lack of trust and respect, school pressure, family demand, punishment, family conflicts, and fears). Similarly, executive functioning is constructed using four manifest variables (congruent hits, incongruent hits, total HDT, and total good choices). The latent variable SEQS is constructed using four manifest variables (support SEQS, stimulation SEQS, structure SEQS, and social integration SEQS). The path connections of the SEM-PLS model are shown in Figure 7.

SEMs, in general, are not designed for testing causal relations among the constructs but provide useful information of the path relations supported by a framework (Cortés, 2019, pp. 113–115).

Additionally, we need to provide some criteria that ensure the model has a good fit. In SEM-PLS, we need to show that there is discriminant validity among the various instruments (Hair et al., 2014).

Once the model parameters have been estimated through the statistical analysis software Smart PLS, the main components of the variables were identified and are listed in Table 3; the average variance extracted (AVE) values greater than 0.50 indicated that environmental quality constructs are valid at a convergent level (Wold, 1985).

The AVE for SEQS is 0.523, indicating that about 52% of the variance is explained by this construct. Similarly, EQSH explains 62.1% of the variance. The composite reliability is similar to Cronbach’s alpha; for example, EQSH has a 0.83 Cronbach’s alpha and 0.86 composite reliability, both numbers indicating good reliability measures. We noticed that EF has the lowest reliability values.

Once the model parameters have been estimated through the statistical analysis software Smart PLS, the main components of the variables were identified and are listed in Table 3; the AVE values greater than 0.50 indicated that environmental quality constructs are valid at a convergent level.

To verify the discriminant validity of the measurement model, we use the Fornell–Larcker criteria (Hair et al., 2014) that compares $\sqrt{\text{AVE}}$ to the correlation between the variables of the model. Table 4 shows along the diagonal the$\sqrt{\text{AVE}}$. For example, $\sqrt{0.523}=0.723$, which is greater than the absolute value of correlation of the latent variables EQSH (0.28), stress (0.18), and EF (0.05). Similarly, the $\sqrt{\text{AVE}}$ for EQSH is $\sqrt{0.621}=0.788$, which is greater than the correlations of EQSH (0.28), stress (0.11), and EF (0.09). Therefore, the discriminant validity is supported (see Table 5).

**TABLE 4:** Quality criteria of the model.

	**AVE**	**Composite reliability**	***R*^2^**	**Cronbach’s alpha**	**Communality**	**Redundancy**
SEQS	0.523	0.810	0	0.746	0	0.523
EQSH	0.621	0.865	0	0.838	0	0.621
Stress	0.416	0.842	0.038	0.803	0.012	0.416
EF	0.339	0.311	0.066	0.375	0.001	0.339

**TABLE 5:** Fornell–Larcker criterion for discriminant validity.

	**SEQS**	**EQSH**	**Stress**	**EF**
SEQS	0.72			
EQSH	0.2824	0.79		
Stress	–0.1846	–0.1129	0.65	
EF	0.0521	–0.0904	–0.2249	0.58

To verify the discriminant validity of the measurement model, the square root of the AVE was compared with the correlation between the variables of the model. When the square root is greater than 0.5 in all cases, it is assumed that the model is valid in a discriminant way (Wold, 1985).

The latent variable stress is formed by lack of acceptance (4.655), verbal aggression (4.080), lack of confidence (3.99), school pressure (3.26), family demand (4.335), punishment (1.60), family problems (3.09), and fear (2.135), while the latent variable of executive functioning is positively shaped by congruent hits (1.602), incongruent successes (1.674), good choices (0.947), and total score (0.098).

The criteria used to evaluate an internal model involve the revision of the coefficient of determination *R*^2^, the standardized trajectory coefficients, the statisticians *t*, and the levels of significance (*p* = 0.05); there are paths that explain a negative impact of housing quality on executive functioning (planning and decision making) (-0.063), while the quality of the school environment has a positive impact (0.012). The mediating role of stress stands out, being negative between the quality of housing (-0.061) and school quality (-0.170), over executive functioning (-0.190).

## Discussion

From the results about the physical characteristics of the school environment, we observe that a warm environment, high-density conditions in the classrooms according to the guidelines (Maxwell, 2006), and the noise levels experienced by the students influence overstimulation, being able to generate overcrowding. Later on, it will be necessary to integrate to the model the perception of the students about this phenomenon.

Some of the main characteristics in housing (Evans et al., 1989) are the type of construction, whether attached or not (it refers to houses that are built at the same time and that share at least one wall); the number of levels or floors; and finally the environmental quality, which in itself implies a broad set of characteristics, from structural quality to the presence of stimulating materials for learning and linguistic stimulation and even the safety of the physical environment (Bradley et al., 1989). It was possible to identify that the quality experienced by the different age groups is present in their homes, with those having low grades experiencing conditions that make it difficult to satisfy an adequate structure, sources of stimulation, and even support from their homes (CONAVI, 2012).

Several studies confirm the influence that environmental deficiencies can have throughout the growth of children, related to low SES (Schmitt and Lipscomb, 2016) or on the contrary that a home without deprivation favors the training of skills and competencies in children, which are maintained throughout adulthood (Evans and Kim, 2012). Therefore it is worrying that the youngest are experiencing these levels of quality in their microsystems. In Mexico, although Article 4 of the Constitution establishes “the right of every family to have a decent and decorous home,” in this article, not even the Housing Law specifies the minimum characteristics it must have; only some criteria formulated by the National Housing Commission (CONAVI) are available for the quality indicator, and housing spaces include two dimensions: the construction material of the home and the sizes.

As was also observed in the PLS model, other environments besides housing that may lag due to lack of resources, in this case, are school environments; those who enter an institution of this type from a very young age are more vulnerable, compared to those who entered at an older age, since they are more likely to recover as they have already consolidated aspects of their growth in other possibly healthier environments such as their homes (Bronfenbrenner and Morris, 2006).

Although classrooms offer the possibility of conversation and interactive activities, as a consequence, noise is generated inevitably, so these activities must be regulated, because high levels of stimulation require higher levels of psychic energy to maintain a certain concentration. The WHO recommends a maximum noise level of 35 dB in schools; however, the lowest noise level in this sample was 53 dB. This phenomenon can eventually lead to attention overload or the so-called cognitive fatigue, which inhibits learning (Estrada, 2007; Galindo and Sheldon, 2012).

In conclusion, it is important to focus efforts to be able to provide spaces of environmental quality for the positive development of children even when housing is important. It is very important that they develop and learn in a school environment that alleviates and generates a positive effect that compensates for their family microsystem (Choi et al., 2016), since intervening with or modifying the family environment with the necessary control is unlikely.

However, the house should also be one of the primary environments to intervene thinking that it represents a model of physical, social, and temporal stability, and ideally, it should not present elements of chaos that obstruct such stability according to numerous investigations (Wenner et al., 1985).

## Data Availability Statement

The datasets generated for this study are available on request to the corresponding author.

## Ethics Statement

Ethical review and approval was not required for the study on human participants in accordance with the local legislation and institutional requirements. Written informed consent to participate in this study was provided by the participants’ legal guardian/next of kin.

## Author Contributions

JC-G, PO-A, and DV contributed conception and design of the study. JC-G and DV organized the database and performed the statistical analysis. JC-G wrote the first draft of the manuscript. JC-G and PO-A wrote sections of the manuscript. All authors contributed to conception and design of the study, manuscript revision, read and approved the submitted version.

## Conflict of Interest

The authors declare that the research was conducted in the absence of any commercial or financial relationships that could be construed as a potential conflict of interest.
